# Supplementary material for: Transcriptomic profiling of tissue environments critical for post-embryonic patterning and morphogenesis of zebrafish skin
Source: eLife. 2023 Sep 11;12:RP86670. doi: 10.7554/eLife.86670 (PMC10495112; doi:10.7554/eLife.86670)
Supplement: Supplementary file 1. [file elife-86670-supp1.docx]

**Published markers of skin and skin-associated cell types**

| **Cell type** | **Marker (citation)** | **Note** |
| --- | --- | --- |
| Basal Cell | *tp63* (Pellegrini et al., 2001), *apoeb* (Grehan et al., 2001) |  |
| Endothelial Cell | *fgd5a* (Cheng et al., 2012) |  |
| Goblet cells | *muc5*.1 (Okuda et al., 2019) (Jevtov et al., 2014) |  |
| Hypodermis | *col6a3* (Gara et al., 2011), *csf1b* (Lang et al., 2009) |  |
| Ionocytes | *kcnd2* (Pan et al., 2022) |  |
| Iridophore | *gpnmb* (Saunders et al., 2019), *pnp4a* (Lang et al., 2009) |  |
| Leukocyte | *ptprc* (CD45) (Antignano et al., 2019) |  |
| Melanophore | *tyrp1b* (Orlow et al., 1993) |  |
| MLC | *adgrg11* (Alemany et al., 2018; Lin et al., 2019) |  |
| Muscle | *neb* (Labeit et al., 2011) |  |
| NaR ionocytes | *atp1b1b* (Lin et al., 2006) |  |
| Pre-SFC | *col6a3* (general dermal marker) (Gara et al., 2011), *runx2b* (Li et al., 2009) | Simplest diagnosis: *col6a3*+, *runx2b*+; *sp7*-. |
| Periderm | *krt4* (Chen et al., 2011) |  |
| pLL hair cell | *myo7aa* (Gibson et al., 1995) |  |
| pLL mantle cell | *fat1b* (Steiner et al., 2014) |  |
| pLL support cell | *slc1a3a* (Lush et al., 2019) |  |
| PN/Schwann cell | *mbpa* (Takahashi et al., 1985) |  |
| Dermal mesenchyme/  Reticulate dermis | *col6a3* (general dermal marker) (Gara et al., 2011), *postnb* (Fibroblast marker) (Crawford et al., 2015) | Simplest diagnosis: *col6a3*+; *postnb*+; *csf1b*- |
| SFC | *sp7* (Aman et al., 2018) |  |
| Subrabasal cell | *cldna* (Hou et al., 2020) |  |
| Xanthophore | *pax7b* (Nord et al., 2016) |  |

**References**

Alemany A, Florescu M, Baron CS, Peterson-Maduro J, van Oudenaarden A. 2018. Whole-organism clone tracing using single-cell sequencing. *Nature* **556**:108-12. doi: 10.1038/nature25969, PMID: 29590089

Aman AJ, Fulbright AN, Parichy DM. 2018. Wnt/beta-catenin regulates an ancient signaling network during zebrafish scale development. *Elife* **7**. doi: 10.7554/eLife.37001, PMID: 30014845

Antignano F, Poon G, Facca V, Separovic L, Valdez Y, Kokaji AI, Woodside SM, Eaves AC, Thomas TE. 2019. Isolation of mouse CD45 positive leukocytes from tissues. *The Journal of Immunology* **202**:130.18-.18.

Chen CF, Chu CY, Chen TH, Lee SJ, Shen CN, Hsiao CD. 2011. Establishment of a transgenic zebrafish line for superficial skin ablation and functional validation of apoptosis modulators in vivo. *PLoS ONE* **6**:e20654. doi: 10.1371/journal.pone.0020654, PMID: 21655190

Cheng C, Haasdijk R, Tempel D, van de Kamp EH, Herpers R, Bos F, Den Dekker WK, Blonden LA, de Jong R, Bürgisser PE, Chrifi I, Biessen EA, Dimmeler S, Schulte-Merker S, Duckers HJ. 2012. Endothelial cell-specific FGD5 involvement in vascular pruning defines neovessel fate in mice. *Circulation* **125**:3142-58. doi: 10.1161/circulationaha.111.064030, PMID: 22661514

Crawford J, Nygard K, Gan BS, O'Gorman DB. 2015. Periostin induces fibroblast proliferation and myofibroblast persistence in hypertrophic scarring. *Exp Dermatol* **24**:120-6. doi: 10.1111/exd.12601, PMID: 25421393

Gara SK, Grumati P, Squarzoni S, Sabatelli P, Urciuolo A, Bonaldo P, Paulsson M, Wagener R. 2011. Differential and restricted expression of novel collagen VI chains in mouse. *Matrix Biology* **30**:248-57. doi: <https://doi.org/10.1016/j.matbio.2011.03.006>,

Gibson F, Walsh J, Mburu P, Varela A, Brown KA, Antonio M, Beisel KW, Steel KP, Brown SD. 1995. A type VII myosin encoded by the mouse deafness gene shaker-1. *Nature* **374**:62-4. doi: 10.1038/374062a0, PMID: 7870172

Grehan S, Allan C, Tse E, Walker D, Taylor JM. 2001. Expression of the Apolipoprotein E Gene in the Skin is Controlled by a Unique Downstream Enhancer. *Journal of Investigative Dermatology* **116**:77-84. doi: <https://doi.org/10.1046/j.1523-1747.2001.00213.x>,

Hou Y, Lee HJ, Chen Y, Ge J, Osman FOI, McAdow AR, Mokalled MH, Johnson SL, Zhao G, Wang T. 2020. Cellular diversity of the regenerating caudal fin. *Sci Adv* **6**:eaba2084. doi: 10.1126/sciadv.aba2084, PMID: 32851162

Jevtov I, Samuelsson T, Yao G, Amsterdam A, Ribbeck K. 2014. Zebrafish as a model to study live mucus physiology. *Sci Rep* **4**:6653. doi: 10.1038/srep06653, PMID: 25323747

Labeit S, Ottenheijm CA, Granzier H. 2011. Nebulin, a major player in muscle health and disease. *Faseb j* **25**:822-9. doi: 10.1096/fj.10-157412, PMID: 21115852

Lang MR, Patterson LB, Gordon TN, Johnson SL, Parichy DM. 2009. Basonuclin-2 requirements for zebrafish adult pigment pattern development and female fertility. *PLoS Genet* **5**:e1000744. doi: 10.1371/journal.pgen.1000744, PMID: 19956727

Li N, Felber K, Elks P, Croucher P, Roehl HH. 2009. Tracking gene expression during zebrafish osteoblast differentiation. *Dev Dyn* **238**:459-66. doi: 10.1002/dvdy.21838, PMID: 19161246

Lin LY, Horng JL, Kunkel JG, Hwang PP. 2006. Proton pump-rich cell secretes acid in skin of zebrafish larvae. *Am J Physiol Cell Physiol* **290**:C371-8. doi: 10.1152/ajpcell.00281.2005, PMID: 16148031

Lin X, Zhou Q, Zhao C, Lin G, Xu J, Wen Z. 2019. An Ectoderm-Derived Myeloid-like Cell Population Functions as Antigen Transporters for Langerhans Cells in Zebrafish Epidermis. *Dev Cell* **49**:605-17.e5. doi: 10.1016/j.devcel.2019.03.028, PMID: 31006648

Lush ME, Diaz DC, Koenecke N, Baek S, Boldt H, St Peter MK, Gaitan-Escudero T, Romero-Carvajal A, Busch-Nentwich EM, Perera AG, Hall KE, Peak A, Haug JS, Piotrowski T. 2019. scRNA-Seq reveals distinct stem cell populations that drive hair cell regeneration after loss of Fgf and Notch signaling. *Elife* **8**. doi: 10.7554/eLife.44431, PMID: 30681411

Nord H, Dennhag N, Muck J, von Hofsten J. 2016. Pax7 is required for establishment of the xanthophore lineage in zebrafish embryos. *Mol Biol Cell* **27**:1853-62. doi: 10.1091/mbc.E15-12-0821, PMID: 27053658

Okuda K, Chen G, Subramani DB, Wolf M, Gilmore RC, Kato T, Radicioni G, Kesimer M, Chua M, Dang H, Livraghi-Butrico A, Ehre C, Doerschuk CM, Randell SH, Matsui H, Nagase T, O'Neal WK, Boucher RC. 2019. Localization of Secretory Mucins MUC5AC and MUC5B in Normal/Healthy Human Airways. *Am J Respir Crit Care Med* **199**:715-27. doi: 10.1164/rccm.201804-0734OC, PMID: 30352166

Orlow SJ, Boissy RE, Moran DJ, Pifko-Hirst S. 1993. Subcellular distribution of tyrosinase and tyrosinase-related protein-1: implications for melanosomal biogenesis. *J Invest Dermatol* **100**:55-64. doi: 10.1111/1523-1747.ep12354138, PMID: 8423398

Pan W, Godoy RS, Cook DP, Scott AL, Nurse CA, Jonz MG. 2022. Single-cell transcriptomic analysis of neuroepithelial cells and other cell types of the gills of zebrafish (Danio rerio) exposed to hypoxia. *Sci Rep* **12**:10144. doi: 10.1038/s41598-022-13693-1, PMID: 35710785

Pellegrini G, Dellambra E, Golisano O, Martinelli E, Fantozzi I, Bondanza S, Ponzin D, McKeon F, De Luca M. 2001. p63 identifies keratinocyte stem cells. *Proc Natl Acad Sci U S A* **98**:3156-61. doi: 10.1073/pnas.061032098, PMID: 11248048

Steiner AB, Kim T, Cabot V, Hudspeth AJ. 2014. Dynamic gene expression by putative hair-cell progenitors during regeneration in the zebrafish lateral line. *Proc Natl Acad Sci U S A* **111**:E1393-401. doi: 10.1073/pnas.1318692111, PMID: 24706895

Takahashi N, Roach A, Teplow DB, Prusiner SB, Hood L. 1985. Cloning and characterization of the myelin basic protein gene from mouse: one gene can encode both 14 kd and 18.5 kd MBPs by alternate use of exons. *Cell* **42**:139-48. doi: <https://doi.org/10.1016/S0092-8674(85)80109-4>,
